# Environment Classification for Robotic Leg Prostheses and Exoskeletons Using Deep Convolutional Neural Networks

**DOI:** 10.3389/fnbot.2021.730965

**Published:** 2022-02-04

**Authors:** Brokoslaw Laschowski, William McNally, Alexander Wong, John McPhee

**Affiliations:** ^1^Department of Systems Design Engineering, University of Waterloo, Waterloo, ON, Canada; ^2^Waterloo Artificial Intelligence Institute, University of Waterloo, Waterloo, ON, Canada

**Keywords:** computer vision, deep learning, exoskeletons, rehabilitation robotics, prosthetics, wearables, artificial intelligence, biomechatronics

## Abstract

Robotic leg prostheses and exoskeletons can provide powered locomotor assistance to older adults and/or persons with physical disabilities. However, the current locomotion mode recognition systems being developed for automated high-level control and decision-making rely on mechanical, inertial, and/or neuromuscular sensors, which inherently have limited prediction horizons (i.e., analogous to walking blindfolded). Inspired by the human vision-locomotor control system, we developed an environment classification system powered by computer vision and deep learning to predict the oncoming walking environments prior to physical interaction, therein allowing for more accurate and robust high-level control decisions. In this study, we first reviewed the development of our “ExoNet” database—the largest and most diverse open-source dataset of wearable camera images of indoor and outdoor real-world walking environments, which were annotated using a hierarchical labeling architecture. We then trained and tested over a dozen state-of-the-art deep convolutional neural networks (CNNs) on the ExoNet database for image classification and automatic feature engineering, including: EfficientNetB0, InceptionV3, MobileNet, MobileNetV2, VGG16, VGG19, Xception, ResNet50, ResNet101, ResNet152, DenseNet121, DenseNet169, and DenseNet201. Finally, we quantitatively compared the benchmarked CNN architectures and their environment classification predictions using an operational metric called “NetScore,” which balances the image classification accuracy with the computational and memory storage requirements (i.e., important for onboard real-time inference with mobile computing devices). Our comparative analyses showed that the EfficientNetB0 network achieves the highest test accuracy; VGG16 the fastest inference time; and MobileNetV2 the best NetScore, which can inform the optimal architecture design or selection depending on the desired performance. Overall, this study provides a large-scale benchmark and reference for next-generation environment classification systems for robotic leg prostheses and exoskeletons.

## Introduction

There are currently hundreds of millions of individuals worldwide with mobility impairments resulting from aging and/or physical disabilities (Grimmer et al., 2019). Fortunately, newly-developed robotic leg prostheses and exoskeletons can replace the propulsive function of amputated or impaired biological limbs and allow users to perform daily locomotor activities that require net positive power generation using motorized hip, knee, and/or ankle joints (Tucker et al., 2015; Young and Ferris, 2017; Laschowski and Andrysek, 2018; Krausz and Hargrove, 2019). However, the control of these wearable robotic devices is extremely difficult and often considered one of the leading challenges to real-world deployment (Tucker et al., 2015; Young and Ferris, 2017).

**Figure 1:**
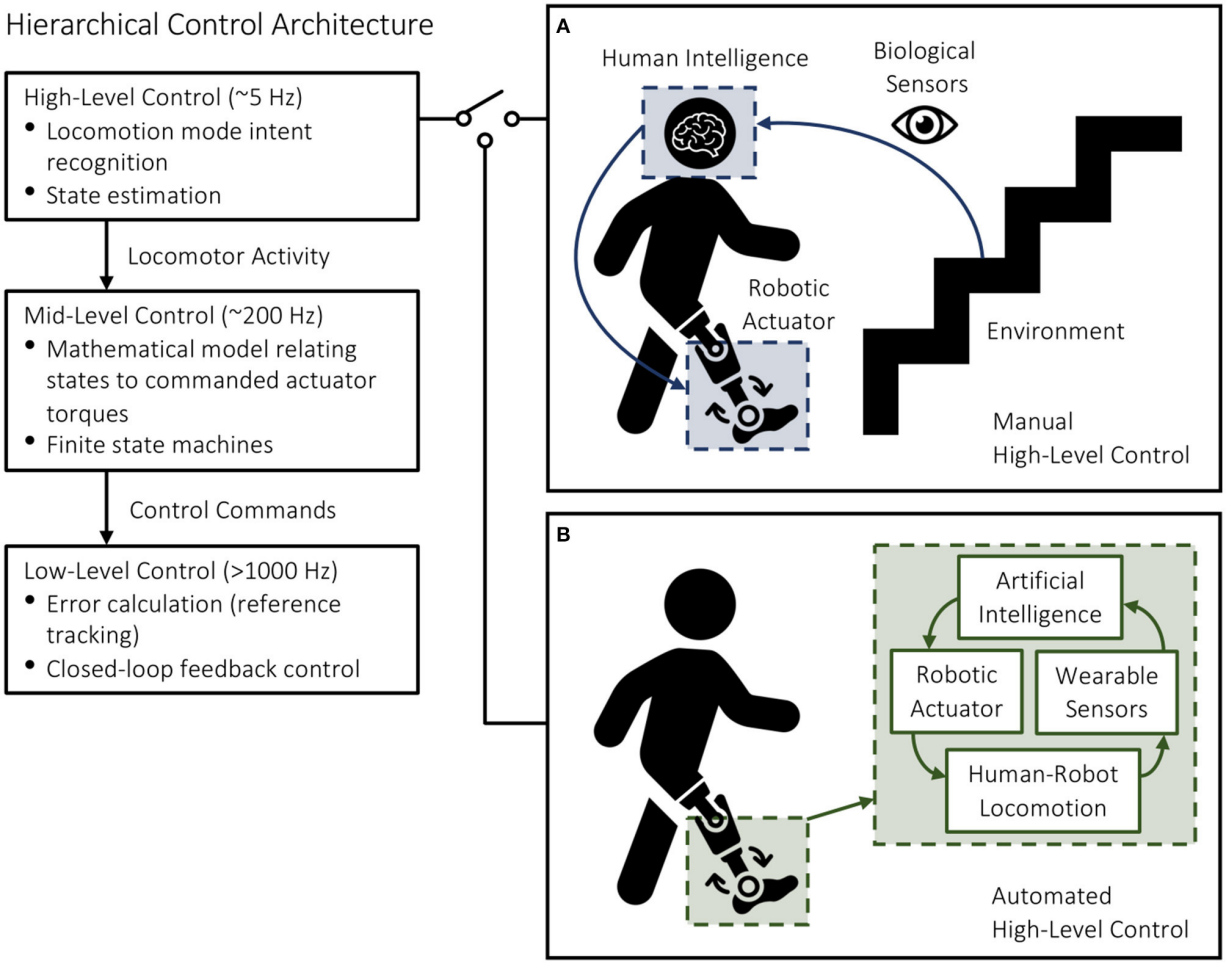
Hierarchical control architecture of robotic leg prostheses and exoskeletons, including high, mid, and low-level controllers. The high-level controller selects the desired locomotion mode using either **(A)** manual communication from the user (i.e., for commercially available devices) or **(B)** automated systems (i.e., for devices under research and development).

Most robotic leg prostheses and exoskeletons use a hierarchical control architecture, including high, mid, and low-level controllers (Tucker et al., 2015; Young and Ferris, 2017) (Figure 1). The high-level controller is responsible for state estimation and predicting the user's locomotor intent. The mid-level controller converts the locomotor activity into mode-specific reference trajectories using dynamic equations of the biomechatronic system; this level of control often consists of individual finite-state machines with discrete mechanical impedance parameters like stiffness and damping coefficients, which are heuristically tuned for different locomotor activities to generate the desired device states. The low-level controller uses standard controls engineering algorithms like proportional-integral-derivative (PID) control to calculate the error between the measured and desired device states and command the robotic actuators to minimize the error *via* reference tracking and closed-loop feedback control (Tucker et al., 2015; Young and Ferris, 2017; Krausz and Hargrove, 2019).

**Figure 2:**
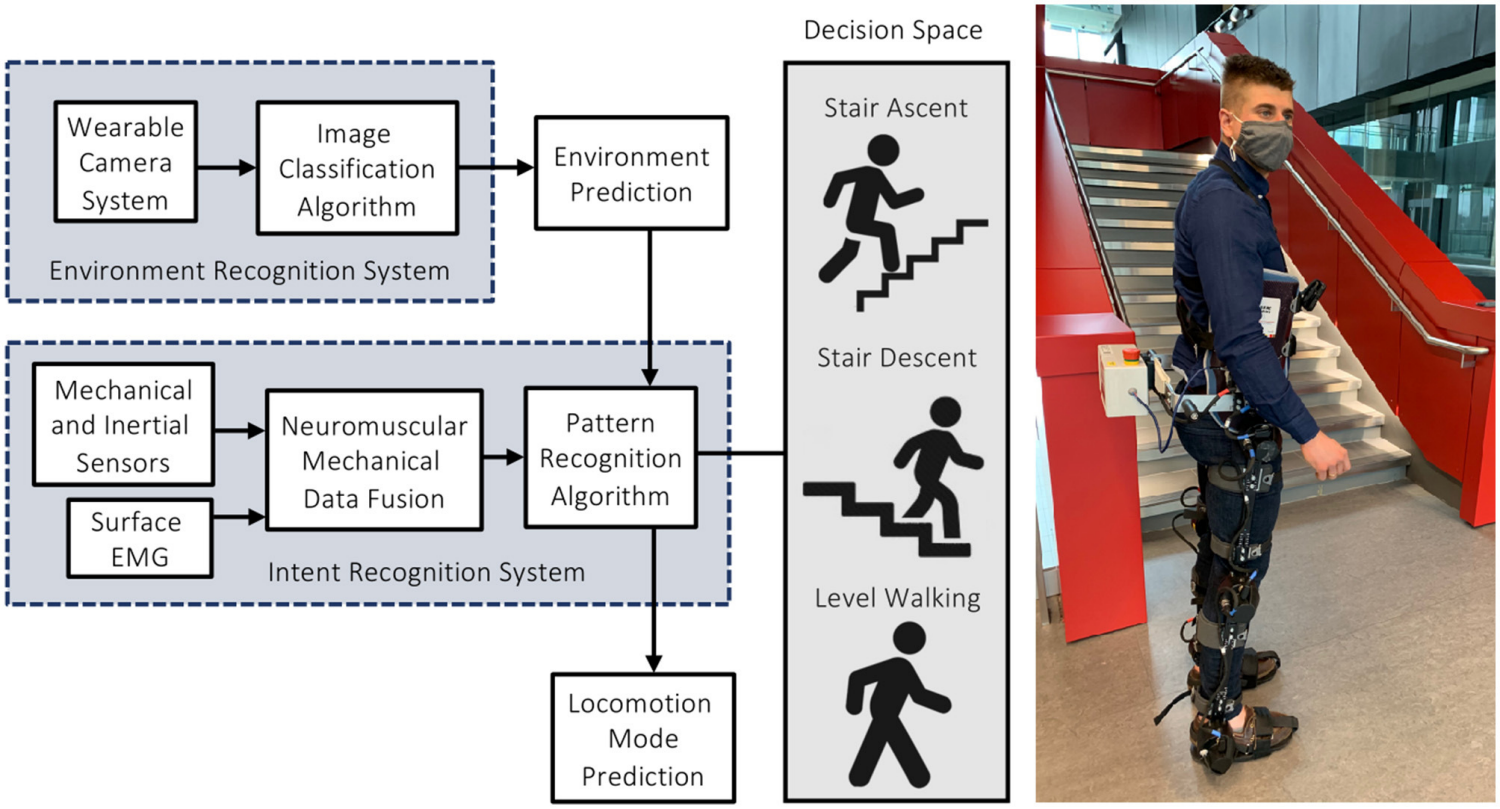
An automated locomotion mode recognition system for robotic leg prostheses and exoskeletons, also known as an intent recognition system or intelligent high-level controller. These systems can be supplemented with an environment recognition system to predict the oncoming walking environments prior to physical interaction, therein minimizing the high-level decision space. The photograph (right) is the lead author wearing our robotic exoskeleton.

High-level transitions between locomotor activities remain a significant challenge. Inaccurate and/or delayed decisions could result in loss-of-balance and injury, which can be especially problematic when involving stairs. Switching between different mid-level controllers is supervised by the high-level controller, which infers the locomotor intent using either sensor data (i.e., for devices under research and development) or direct communication from the user (i.e., for commercially available devices) (Figure 1). For instance, the Össur Power Knee prosthesis and the ReWalk and Indego powered exoskeletons require the users to perform exaggerated movements or use hand controls to manually switch between locomotion modes (Tucker et al., 2015; Young and Ferris, 2017). Although highly accurate in communicating the user's locomotor intent, manual high-level control and decision making can be time-consuming, inconvenient, and cognitively demanding (Karacan et al., 2020). Researchers have thus been working on developing automated locomotion mode recognition systems using pattern recognition algorithms and data from wearable sensors like inertial measurement units (IMUs) and surface electromyography (EMG), therein shifting the high-level control burden from the user to an intelligent controller (Figure 2).

Mechanical sensors embedded in robotic leg prostheses and exoskeletons can be used for state estimation by measuring the joint angles and angular velocities, and interaction forces and/or torques between the human and device, and between the device and environment. IMU sensors can measure angular velocities, accelerations, and direction of body segments. Although mechanical and inertial sensors can allow for fully integrated control systems, these sensors only respond to the user's movements. In contrast, the electrical potentials of biological muscles, as recorded using surface EMG, precede movement initiation and thus could predict locomotion mode transitions with small prediction horizons. EMG signals could also be used for proportional myoelectric control (Nasr et al., 2021). Fusing information from mechanical and/or inertial sensors with surface EMG, known as neuromuscular-mechanical data fusion, can improve the locomotion mode recognition accuracies and decision times compared to implementing either system individually (Huang et al., 2011a,b; Du et al., 2012; Wang et al., 2013; Liu et al., 2016; Krausz and Hargrove, 2021). However, neuromuscular-mechanical data are user-dependent, therein often requiring time-consuming experiments to amass individual datasets, and surface EMG require calibration and are susceptible to fatigue, changes in electrode-skin conductivity, and crosstalk between adjacent muscles (Tucker et al., 2015; Young and Ferris, 2017). Despite the advances in automated intent recognition using mechanical, inertial, and/or neuromuscular sensors, further improvements in the system performance are desired for safe and robust locomotor control.

## Literature Review

Taking inspiration from the human vision-locomotor control system, supplementing neuromuscular-mechanical data with information about the oncoming walking environment could improve the automated high-level control performance (Figure 2). Environment sensing would precede modulation of the user's muscle activations and/or walking biomechanics, therein allowing for more accurate and robust high-level control decisions by minimizing the decision space. During human locomotion, the central nervous system acquires state information from biological sensors (e.g., the eyes) through ascending pathways, which are used to actuate and control the musculoskeletal system through feedforward efferent commands (Patla, 1997; Tucker et al., 2015). However, these control loops are compromised in persons using assistive devices due to limitations in the human-machine data communication. Environment sensing and classification could artificially restore these control loops for automated high-level control. Environment information could also be used to adapt the mid-level reference trajectories (e.g., increasing the actuator joint torques for toe clearance corresponding to an obstacle height) (Zhang et al., 2020); optimal path planning (e.g., identifying opportunities for energy recovery) (Laschowski et al., 2019a, 2021a); and varying foot placement based on the walking surface (Leo and Farinella, 2018).

One of the earliest studies fusing neuromuscular-mechanical data with environment information for prosthetic leg control came from Zhang et al. (2011), Huang et al. (2011a), Du et al. (2012), Wang et al. (2013), Liu et al. (2016),. Different walking environments were statistically modeled as prior probabilities using the principle of maximum entropy and incorporated into the discriminant function of an LDA classification algorithm. The group simulated different walking environments by adjusting the prior probabilities of each class, which allowed their locomotion mode recognition system to adapt to different environments. Using these adaptive prior probabilities based on terrain information significantly outperformed (i.e., 95.5% classification accuracy) their locomotion mode recognition system based on neuromuscular-mechanical data alone with equal prior probabilities (i.e., 90.6% accuracy) (Wang et al., 2013). These seminal papers showed (1) how environment information could be incorporated into an automated high-level controller; (2) that including such information could improve the locomotion mode recognition accuracies and decision times; and (3) that the controller could be relatively robust to noisy and imperfect environment predictions such that the neuromuscular-mechanical data dominated the high-level decision making (Huang et al., 2011a; Du et al., 2012; Wang et al., 2013; Liu et al., 2016).

Several researchers have explored using wearable radar detectors (Kleiner et al., 2018) and laser rangefinders (Zhang et al., 2011; Wang et al., 2013; Liu et al., 2016) for active environment sensing. Unlike camera-based systems, these sensors circumvent the need for computationally expensive image processing and classification. Radar can measure distances through non-conducting materials like clothing and are invariant to outdoor lighting conditions and surface textures. Using a leg-mounted radar detector, Herr's research group (Kleiner et al., 2018) measured stair distances and heights within 1.5 cm and 0.34 cm average accuracies, respectively, up to 6.25 m maximum distances. However, radar reflection signatures struggle with source separation of multiple objects and have relatively low resolution. Huang and colleagues (Zhang et al., 2011; Wang et al., 2013; Liu et al., 2016) developed a waist-mounted system with an IMU and laser rangefinder to reconstruct the geometry of the oncoming walking environments between 300 and 10,000 mm ranges. Environmental features like the terrain height, distance, and slope were used for classification *via* heuristic rule-based thresholds. The system achieved 98.1% classification accuracy (Zhang et al., 2011). While simple and effective, their system required subject-specific calibration (e.g., the device mounting height) and provided only a single distance measurement.

**Figure 3:**
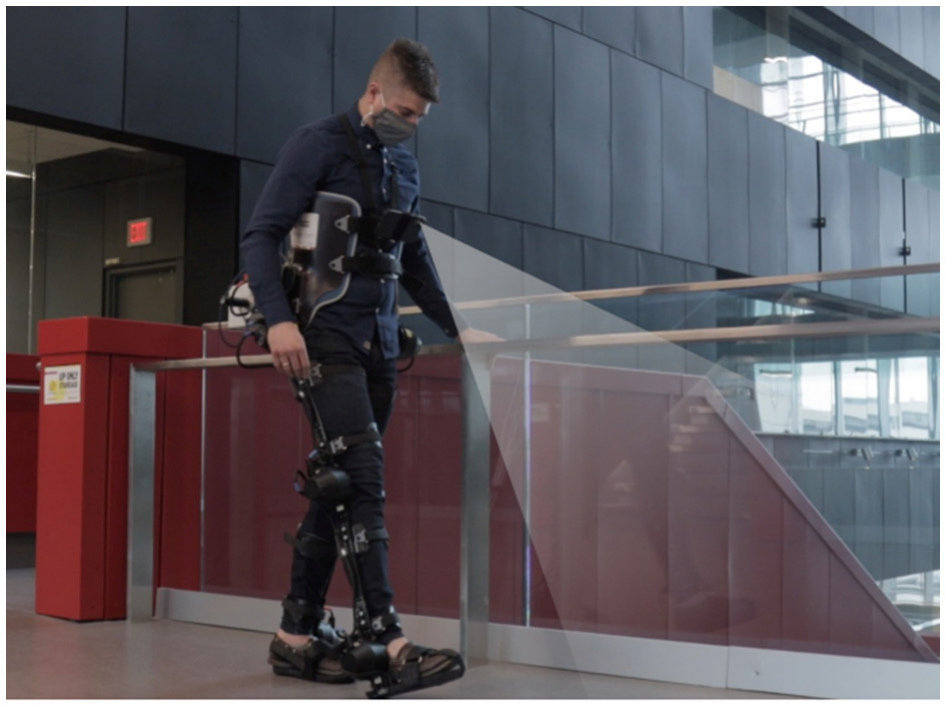
Photograph of the lead author walking with our robotic exoskeleton with vision-based environment sensing superimposed.

Compared to radar and laser rangefinders, cameras can provide more detailed information about the field-of-view and detect physical obstacles and terrain changes in peripheral locations (Figure 3). Most environment recognition systems have used RGB cameras (Krausz and Hargrove, 2015; Diaz et al., 2018; Khademi and Simon, 2019; Laschowski et al., 2019b, 2020b, 2021b; Novo-Torres et al., 2019; Da Silva et al., 2020; Zhong et al., 2020) or 3D depth cameras (Krausz et al., 2015, 2019; Varol and Massalin, 2016; Hu et al., 2018; Massalin et al., 2018; Zhang et al., 2019b,c,d, 2020; Krausz and Hargrove, 2021; Tschiedel et al., 2021) mounted on the chest (Krausz et al., 2015; Laschowski et al., 2019b, 2020b, 2021b), waist (Khademi and Simon, 2019; Krausz et al., 2019; Zhang et al., 2019d; Krausz and Hargrove, 2021), or lower-limbs (Varol and Massalin, 2016; Diaz et al., 2018; Massalin et al., 2018; Zhang et al., 2019b,c, 2020; Da Silva et al., 2020; Zhong et al., 2020) (Table 1). Few studies have adopted head-mounted cameras for biomimicry (Novo-Torres et al., 2019; Zhong et al., 2020). Zhong et al. (2020) recently compared the effects of different wearable camera positions on classification performance. Compared to glasses, their leg-mounted camera more accurately detected closer walking environments but struggled with incline stairs, often capturing only 1–2 steps. Although the glasses could detect further-away environments, the head-mounted camera also captured irrelevant features like the sky, which reduced the classification accuracy. The glasses also struggled with detecting decline stairs and had larger standard deviations in classification predictions due to head movement.

**Table 1:** Experimental datasets used for image classification of walking environments for robotic leg prostheses and exoskeletons.

**Reference**	**Camera**	**Body position**	**Dataset size**	**Image resolution**	**Classes**
Da Silva et al. (2020)	RGB	Lower-limb	3,992	512 × 512	6
Diaz et al. (2018)	RGB	Lower-limb	3,992	1,080 × 1,920	6
Khademi and Simon (2019)	RGB	Waist	7,284	224 × 224	3
Krausz and Hargrove (2015)	RGB	Head	5	928 × 620	2
Krausz et al. (2015)	Depth	Chest	170	80 × 60	2
Krausz et al. (2019)	Depth	Waist	4,000	171 × 224	5
Laschowski et al. (2019b)	RGB	Chest	34,254	224 × 224	3
Laschowski et al. (2020b)	RGB	Chest	922,790	1280 × 720	12
Massalin et al. (2018)	Depth	Lower-limb	402,403	320 × 240	5
Novo-Torres et al. (2019)	RGB	Head	40,743	128 × 128	2
Varol and Massalin (2016)	Depth	Lower-limb	22,932	320 × 240	5
Zhang et al. (2019b,c)	Depth	Lower-limb	7,500	224 × 171	5
Zhang et al. (2019d)	Depth	Waist	4,016	2048-point cloud	3
Zhang et al. (2020)	Depth	Lower-limb	7,500	100 × 100	5
Zhong et al. (2020)	RGB	Head and lower-limb	327,000	1,240 × 1,080	6

*Note that the ExoNet database was published in Laschowski et al. (2020b)*.

For image classification, researchers have traditionally used statistical pattern recognition and machine learning algorithms like support vector machines, which require hand-engineering (Krausz et al., 2015, 2019; Varol and Massalin, 2016; Diaz et al., 2018; Hu et al., 2018; Massalin et al., 2018; Da Silva et al., 2020; Krausz and Hargrove, 2021) (Table 2). Hargrove's research group (Krausz et al., 2015, 2019) used standard image processing and rule-based thresholds to detect convex and concave edges and vertical and horizontal planes for stair recognition. Although their algorithm achieved 98.8% classification accuracy, the computations were time-consuming (i.e., ~8 seconds/frame) and the system was evaluated using only five images. In another example, Huang and colleagues (Diaz et al., 2018) achieved 86% image classification accuracy across six environment classes using SURF features and a bag-of-words classifier. Varol's research group used support vector machines for classifying depth images, which mapped extracted features into a high-dimensional space and separated samples into different classes by constructing optimal hyperplanes with maximum margins (Varol and Massalin, 2016; Massalin et al., 2018). Their system achieved 94.1% classification accuracy across five locomotion modes using a cubic kernel SVM and no dimension reduction (Massalin et al., 2018). The average computation time was ~14.9 ms per image. Although SVMs are effective in high-dimensional space and offer good generalization (i.e., robustness to overfitting), these algorithms require manual selection of kernel functions and statistical features, which can be time-consuming and suboptimal.

**Table 2:** Previous environment recognition systems that used heuristics, statistical pattern recognition, or support vector machines for image classification of walking environments.

**Reference**	**Feature extractor and classifier**	**Computing devices**	**Test accuracy (%)**	**Computation time (ms)**
Da Silva et al. (2020)	Local binary pattern and random forest	NVIDIA Jetson TX2	90.0	200
Diaz et al. (2018)	SURF features and bag-of-words model	Intel Core i7-2600 CPU (3.40GHz)	86.0	N/A
Krausz and Hargrove (2015)	Hough transform with Gabor filter or canny edge detector	Intel Core i5	N/A	8000
Krausz et al. (2015)	Heuristic thresholding and edge detector	Intel Core i5	98.8	200
Krausz et al. (2019)	Regions-of-interest and linear discriminate analysis	Intel Core i7-8750H (2.2GHz)	N/A	N/A
Massalin et al. (2018)	Cubic kernel support vector machine	Intel Core i7-2640M (2.8GHz)	94.1	14.9
Varol and Massalin (2016)	Support vector machine	Intel Core i7-2640M (2.8GHz)	99.0	14.9

*Note that each classifier was developed and tested on different image datasets (see Table 1). The computation times are reported per image*.

The latest generation of environment recognition systems has used convolutional neural networks (CNNs) for image classification (Rai and Rombokas, 2018; Khademi and Simon, 2019; Laschowski et al., 2019b, 2021b; Novo-Torres et al., 2019; Zhang et al., 2019b,c,d, 2020; Zhong et al., 2020) (Table 3). Deep learning replaces manually extracted features with multilayer networks that can automatically and efficiently learn the optimal image features from training data. One of the earliest studies came from Laschowski et al. (2019b), who designed and trained a 10-layer convolutional neural network using five-fold cross-validation, which differentiated between three environment classes with 94.9% classification accuracy. The convolutional neural network from Simon's research group (Khademi and Simon, 2019) achieved 99% classification accuracy across three environment classes using transfer learning of pretrained weights. Although CNNs typically outperform SVMs for image classification and bypass the need for manual feature engineering (LeCun et al., 2015), deep learning requires significant and diverse training data to prevent overfitting and promote generalization. The lack of an open-source, large-scale image dataset of walking environments has impeded the development of environment-adaptive control systems for robotic leg prostheses and exoskeletons. To date, researchers each individually collected training data to develop their image classification algorithms. These repetitive measurements are time-consuming and inefficient, and individual private datasets have prevented direct comparisons between classification algorithms from different researchers (Laschowski et al., 2020a).

**Table 3:** Previous environment recognition systems that used convolutional neural networks for image classification of walking environments.

**Reference**	**Operations (billions)**	**Parameters (millions)**	**Computing Devices**	**Test Accuracy (%)**	**Inference Time (ms)**
Khademi and Simon (2019)	7.7	27	Titan X	99.6	50
Laschowski et al. (2019b)	1.2850	4.73	TITAN Xp	94.9	0.9
Novo-Torres et al. (2019)	0.0011	1.13	Geforce GTX 965M	90.0	5.5
Zhang et al. (2019b)	0.0130	0.22	GeForce GTX 1050 Ti	96.8	3.1
Zhang et al. (2019c)	0.0130	0.22	Quadro P400	98.9	3.0
Zhang et al. (2019d)	0.0215	0.05	GeForce GTX 1050 Ti	98.0	2.0
Zhang et al. (2020)	0.0130	0.22	Quadro P400	96.0	3.0
Zhong et al. (2020)^†^	0.0544	2.20	Jetson TX2	95.4	12.7

*Note that each network was trained and tested on different image datasets (see Table 1). The computing hardware were all developed and manufactured by NVIDIA. The number of operations is expressed in multiply-accumulates. The inference times are reported per image.*

*^†^used MobileNetV2 for feature extraction and a Bayesian neural network (gated recurrent unit) for the environment classification*.

Motivated by these limitations, our research group has been developing large-scale environment sensing and classification systems powered by computer vision and deep learning. To support this initiative, we recently published the “ExoNet” database—the largest and most diverse open-source dataset of wearable camera images of real-world walking environments (Laschowski et al., 2020b). In the current study, we first review the development of our ExoNet database (section Environment Sensing). We then made the following original contributions: (1) trained and tested over a dozen state-of-the-art deep convolutional neural networks on the ExoNet database for image classification and automatic feature engineering (section Environment Classification); and (2) quantitatively compared the benchmarked CNN architectures and their environment classification predictions using an operational metric called “NetScore,” which balances the image classification accuracy with the computational and memory storage requirements (i.e., important for onboard real-time inference with mobile computing devices) (section NetScore Evaluations). Overall, this study provides a large-scale benchmark and reference for next-generation environment classification systems for robotic leg prostheses and exoskeletons.

## Materials and Methods

### Environment Sensing

**Figure 4:**
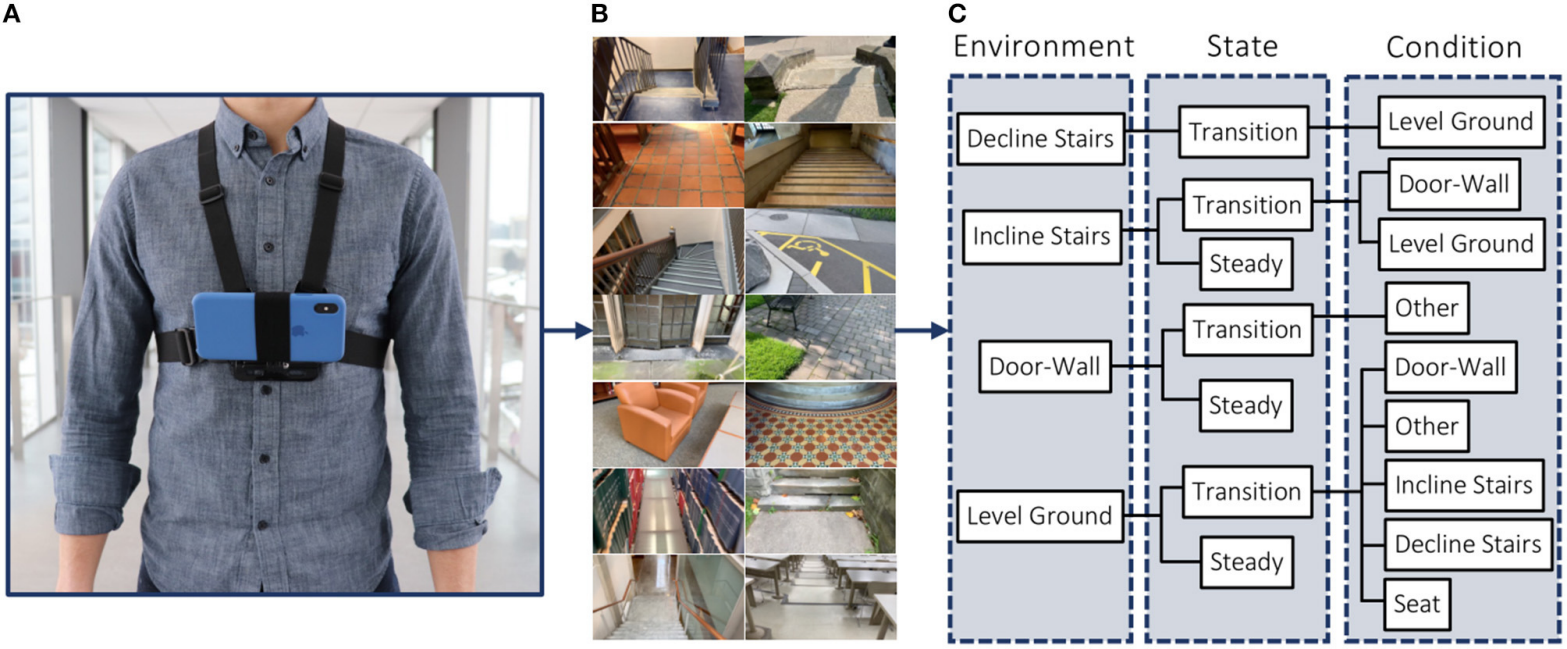
Development of the “ExoNet” database, including **(A)** a photograph of the wearable camera system used for large-scale data collection; **(B)** examples of the high-resolution RGB images of walking environments; and **(C)** a schematic of the 12-class hierarchical labeling architecture.

In this section, we review the development of our ExoNet database (Laschowski et al., 2020b). One subject (sex: male; weight: 77 kg; height: 1.8 m; age: 30 years) was instrumented with a wearable smartphone camera system (iPhone XS Max) (Figure 4). Compared to lower-limb systems (Zhang et al., 2011, 2019b,c, 2020; Varol and Massalin, 2016; Diaz et al., 2018; Hu et al., 2018; Kleiner et al., 2018; Massalin et al., 2018; Rai and Rombokas, 2018; Da Silva et al., 2020), our chest-mounted camera can provide more stable video recording and allow users to wear pants and dresses without obstructing the visual field-of-view. The chest-mount height was ~1.3 m from the ground when the participant stood upright. The smartphone weighs 0.21 kg and has an onboard rechargeable lithium-ion battery, 512-GB of memory storage, and a 64-bit ARM-based integrated circuit (Apple A12 Bionic) with a six-core CPU and four-core GPU; these hardware specifications can theoretically support onboard deep learning inference for real-time environment classification. The relatively lightweight and unobtrusive nature of the wearable camera system allowed for unimpeded human locomotion. Ethical review and approval were not required for this research study in accordance with the University of Waterloo Office of Research Ethics.

**Figure 5:**
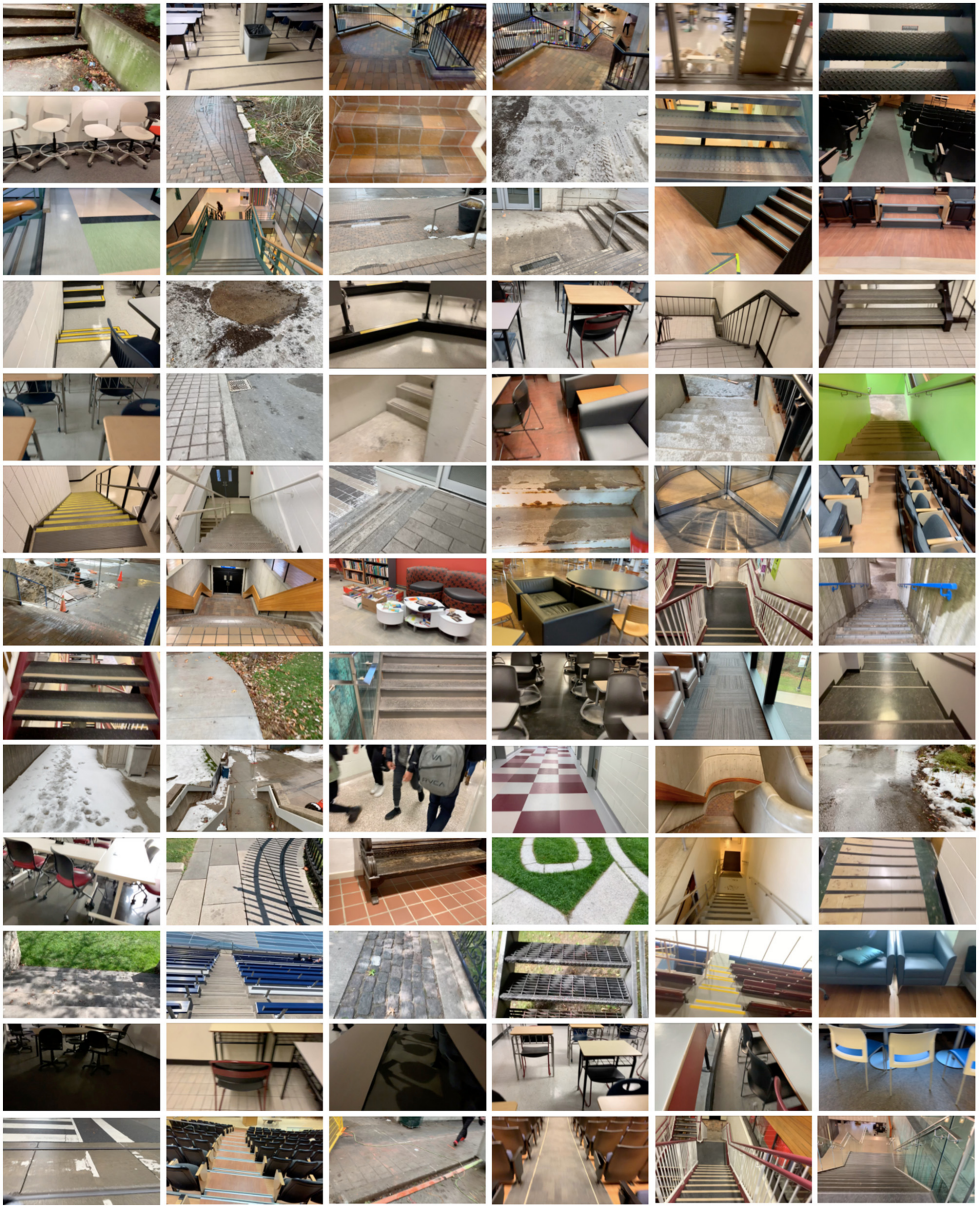
Examples of the wearable camera images of indoor and outdoor real-world walking environments in the ExoNet database. Images were collected at various times throughout the day and across different seasons (i.e., summer, fall, and winter).

Whereas most environment recognition systems have been limited to controlled indoor environments and/or prearranged walking circuits (Zhang et al., 2011, 2019b,c,d; Du et al., 2012; Wang et al., 2013; Krausz et al., 2015, 2019; Liu et al., 2016; Hu et al., 2018; Kleiner et al., 2018; Rai and Rombokas, 2018; Khademi and Simon, 2019; Krausz and Hargrove, 2021), our participant walked around unknown outdoor and indoor real-world environments while collecting images with occlusions and intraclass variations (Figure 5). We collected data at various times throughout the day to include different lighting conditions. Similar to human gaze fixation during walking (Li et al., 2019), the visual field-of-view was 1–5 m ahead of the participant, thereby showing the oncoming walking environment rather than the ground directly underneath the subject's feet. Images were sampled at 30 Hz with 1280 × 720 resolution. We recorded over 52 h of video, amounting to ~5.6 million images. The same environment was never sampled twice to maximize diversity in the dataset. Images were collected during the summer, fall, and winter seasons to capture different weathered surfaces like snow, grass, and multicolored leaves. The image database, which we named ExoNet, was uploaded to the IEEE DataPort repository and is publicly available for download (Laschowski et al., 2020b) at https://ieee-dataport.org/open-access/exonet-database-wearable-camera-images-human-locomotion-environments. The size of the video files is ~140 GB.

For the subject's self-selected walking speed, there were relatively minimal differences between consecutive images sampled at 30 Hz. We therefore downsampled and labeled the images at 5 frames/second to minimize the demands of manual annotation and increase the diversity in image appearances. Similar to the ImageNet dataset (Deng et al., 2009), the ExoNet database was human-annotated using a hierarchical labeling architecture. Images were mainly labeled according to common high-level locomotion modes of robotic leg prostheses and exoskeletons, rather than a purely computer vision perspective. For instance, images of level-ground terrain showing either pavement or grass were not differentiated since both surface textures would be assigned the same high-level locomotion mode (i.e., level-ground walking). However, with advances in control system designs, image classification of different walking surface textures could be beneficial (e.g., adapting the impedance control parameters online for different surface compliances).

Approximately 923,000 images were annotated using a novel 12-class hierarchical labeling architecture (Figure 4; Table 4). The dataset included: 31,628 images of “incline stairs transition wall/door” (I-T-W); 11,040 images of “incline stairs transition level-ground” (I-T-L); 17,358 images of “incline stairs steady” (I-S); 28,677 images of “decline stairs transition level-ground” (D-T-L); 19,150 images of “wall/door transition other” (W-T-O); 36,710 images of “wall/door steady” (W-S); 379,199 images of “level-ground transition wall/door” (L-T-W); 153,263 images of “level-ground transition other” (L-T-O); 26,067 images of “level-ground transition incline stairs” (L-T-I); 22,607 images of “level-ground transition decline stairs” (L-T-D); 119,515 images of “level-ground transition seats” (L-T-E); and 77,576 images of “level-ground steady” (L-S). These class labels were chosen and assigned *post hoc* to capture the different walking environments from the data collection. Similar to (Zhang et al., 2011; Wang et al., 2013; Liu et al., 2016), we included an “other” class to maintain the image classification performance when unlabeled environments and/or objects like pedestrians, cars, and bicycles were observable. Accordingly, the W-T-O and W-S classes include obstructed fields-of-view from walls, doors, and other close-up objects and/or environments.

**Table 4:** The class distributions in the ExoNet database, the images of which were annotated using a hierarchical labeling architecture.

**Label**	**Number of images**	**Percent of dataset (%)**
L-T-W	379,199	41.1
L-T-O	153,263	16.6
L-T-E	119,515	13.0
L-S	77,576	8.4
W-S	36,710	4.0
I-T-W	31,628	3.4
D-T-L	28,677	3.1
L-T-I	26,067	2.8
L-T-D	26,067	2.4
W-T-O	19,150	2.1
I-S	17,358	1.9
I-T-L	11,040	1.2
Total	922,790	100

*A description of the class labels is provided in the text*.

**Figure 6:**
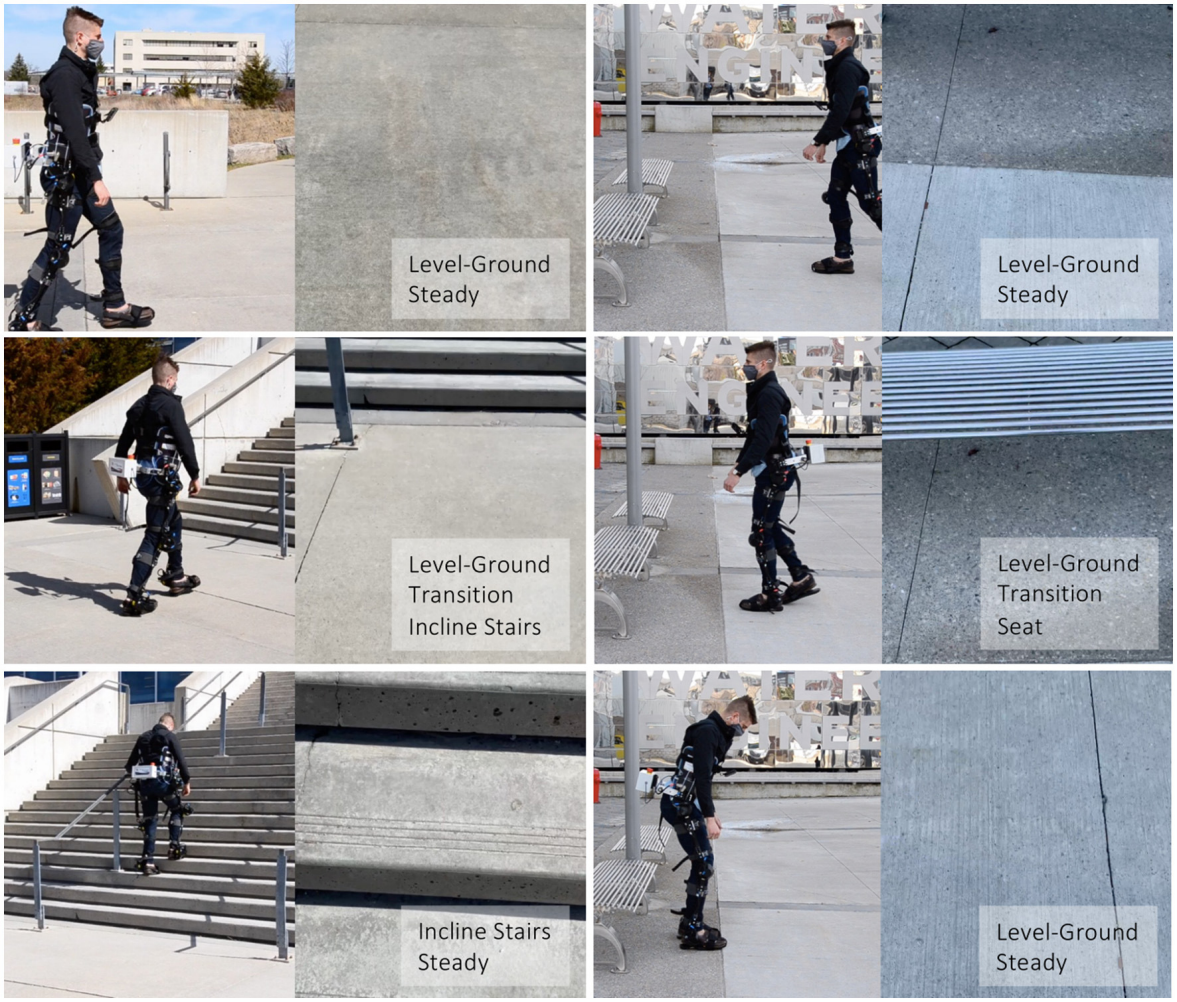
Examples of both “steady” and “transition” states in the ExoNet hierarchical labeling architecture. The top and bottom rows are labeled as steady states and the middle row is labeled a transition state. For each column, the left images show the lead author walking with our robotic exoskeleton and the right images show the concurrent field-of-view of the wearable camera system (i.e., what the exoskeleton sees).

Taking inspiration from Huang et al. (2011a), Du et al. (2012), Wang et al. (2013), Liu et al. (2016), Khademi and Simon (2019), our labeling architecture included both “steady” (S) and “transition” (T) states (Figure 6). A steady state describes an environment where an exoskeleton or prosthesis user would continue to perform the same locomotion mode (e.g., an image showing only level-ground terrain). In contrast, a transition state describes an environment where an exoskeleton or prosthesis high-level controller might switch between locomotion modes (e.g., an image showing both level-ground terrain and incline stairs). Manually labeling these transition states was relatively subjective. For instance, an image showing level-ground terrain was labeled “level-ground transition incline stairs” (L-T-I) when an incline staircase was approximately within the visual field-of-view. Although the ExoNet database was labeled by one designated researcher, consistently determining the exact video frame where an environment would switch between steady and transition states was challenging; Huang et al. (2011a) reported experiencing similar difficulties.

### Environment Classification

Here we describe the design and training of the convolutional neural networks used for image classification and automatic feature engineering. Generally speaking, the CNN architectures contain multiple stacked convolutional and pooling layers with decreasing spatial resolutions and increasing number of feature maps. Starting with an input image, the convolutional layers perform convolution operations (i.e., dot products) between the inputs and convolutional filters. The first few layers extract relatively general features, like edges, while deeper layers learn more abstract, problem-dependent features. The resulting feature maps are passed through a nonlinear activation function. The pooling layers spatially downsample the feature maps to reduce the computational effort by aggregating neighboring elements using either maximum or average values. The architectures conclude with one or more fully connected layers and a loss function, which estimates the probability distribution (i.e., scores) of each labeled class. Like ourselves, most previous CNN-based environment classification systems (Table 3) have used supervised learning such that the differences between the predicted and labeled class scores are calculated and the learnable network parameters (i.e., weights) are optimized to minimize the loss function *via* backpropagation and stochastic gradient descent. After training, the CNNs perform inference on previously unseen data to evaluate the generalizability of the learned parameters.

We used TensorFlow 2.3 and the Keras functional API to build, train, and test over a dozen deep convolutional neutral networks on the ExoNet database, including: EfficientNetB0 (Tan and Le, 2019); InceptionV3 (Szegedy et al., 2015); MobileNet (Howard et al., 2017); MobileNetV2 (Sandler et al., 2018); VGG16 and VGG19 (Simonyan and Zisserman, 2014); Xception (Chollet, 2016); ResNet50, ResNet101, and ResNet152 (He et al., 2015); and DenseNet121, DenseNet169, and DenseNet201 (Huang et al., 2017). These architectures were chosen because they have achieved state-of-the-art image classification performance on other large-scale datasets like ImageNet and are often used for comparative analyses in computer vision (Canziani et al., 2016). During data preprocessing, the images were cropped to an aspect ratio of 1:1 and downsampled to 256 × 256 using bilinear interpolation. Random crops of 224 × 224 were used as inputs to the neural networks; this method of data augmentation helped further increase the sample diversity. The final densely connected layer of each CNN architecture was modified by setting the number of output channels equal to the number of environment classes in the ExoNet database (*n* = 12). We used a softmax loss function to predict the individual class scores. The labeled ExoNet images were split into training (89.5%), validation (3.5%), and testing (7%) sets, the proportions of which are consistent with ImageNet (Deng et al., 2009), which is of comparable size. We experimented with transfer learning of pretrained weights from ImageNet but found no additional performance benefit.

Dropout regularization was applied before the final dense layer to prevent overfitting during training such that the learnable weights were randomly dropped (i.e., activations set to zero) during each forward pass at a rate of 0.5. Images were also randomly flipped horizontally during training to increase stochasticity and promote generalization. We trained each CNN architecture for 40 epochs using a batch size and initial learning rate of 128 and 0.001, respectively; these hyperparameters were experimentally tuned on the validation set. We explored different combinations of batch sizes of 32, 64, 128, and 256; epochs of 20, 40, and 60; dropout rates of 0, 0.2, 0.5; and initial learning rates of 0.01, 0.001, 0.0001, and 0.00001. The learning rate was reduced during training using a cosine weight decay schedule (Loshchilov and Hutter, 2016). We calculated the sparse categorical cross-entropy loss between the labeled and predicted classes and used the Adam optimizer (Kingma and Ba, 2015), which computes backpropagated gradients using momentum and an adaptive learning rate, to update the learnable weights and minimize the loss function. During testing, we used a single central crop of 224 × 224. Training and inference were both performed on a Tensor Processing Unit (TPU) version 3–8 by Google Cloud; these customized chips can allow for accelerated CNN computations (i.e., matrix multiplications and additions) compared to more traditional computing devices.

### NetScore Evaluations

Here we describe our “NetScore” evaluations. The development of deep convolutional neural networks has traditionally focused on improving classification accuracy, often leading to more accurate yet inefficient algorithms with greater computational and memory storage requirements (Canziani et al., 2016). These design features can be especially problematic for deployment on mobile and embedded systems, which inherently have limited operating resources. Despite advances in computing devices like graphics processing units (GPUs), the current embedded systems in robotic leg prostheses and exoskeletons would struggle to support the architectural and computational complexities typically associated with deep learning for computer vision. To facilitate onboard real-time inference, the ideal convolutional neural network would achieve high classification accuracy with minimal parameters, computing operations, and inference time. Motivated by these design principles, we quantitatively evaluated and compared the benchmarked CNN architectures (N) and their environment classification predictions on the ExoNet database using an operational metric called “NetScore” (Wong, 2018):


(1) $$\begin{array}{l} \Omega \left(N\right)=20\log\left(\frac{a{\left(N\right)}^{\alpha }}{p{\left(N\right)}^{\beta }m{\left(N\right)}^{\gamma }}\right) \end{array}$$

where a(N) is the image classification accuracy during inference (0–100%), p(N) is the number of parameters expressed in millions, m(N) is the number of multiply–accumulates expressed in billions, and α, β, and γ are coefficients that control the effects of the classification accuracy, and the architectural and computational complexities on the NetScore (Ω), respectively. We set the coefficients to {α = 2;β = 0.5;γ = 0.5} to better emphasize the classification accuracy while partially considering the parameters and computing operations since neural networks with low accuracy are less practical, regardless of the size and speed. Note that the NetScore does not explicitly account for inference time. The number of parameters p(N) and multiply–accumulates m(N) are assumed to be representative of the architectural and computational complexities, respectively, both of which are inversely proportional to the NetScore.

## Results

Table 5 summarizes the benchmarked CNN architectures (i.e., number of parameters and computing operations) and their environment classification performances on the ExoNet database (i.e., prediction accuracies, inference times, and NetScores). The EfficientNetB0 network achieved the highest image classification accuracy (*C*_*a*_) during inference (73.2% accuracy), that being the percentage of true positives (47,265 images) out of the total number of images in the testing set (64,568 images) $\left(C_{a}=\frac{\mathrm{True}\;\mathrm{Positives}}{\mathrm{Total}\;\mathrm{Images}}\times 100\%\right)$. In contrast, the VGG19 network produced the least accurate predictions, with an overall image classification accuracy of 69.2%. The range of accuracies across the benchmarked CNN architectures was thus relatively small (i.e., maximum arithmetic difference of 4 percentage points). We observed relatively weak statistical correlations between both the number of parameters (Pearson *r* = −0.3) and computing operations (Pearson *r* = −0.59) and the classification accuracies on the ExoNet database across the benchmarked CNN architectures.

**Table 5:** The benchmarked CNN architectures and their environment classification performances during inference on the ExoNet database.

**CNN architecture**	**Operations (GMACs)**	**Parameters (M)**	**test accuracy (%)**	**Inference time (ms)**	**NetScore**
EfficientNetB0	0.39	4.06	**73.2**	2.5	72.6
InceptionV3	2.84	21.83	71.9	4.1	56.3
MobileNet	0.57	3.24	71.1	1.6	71.4
MobileNetV2	**0.30**	**2.27**	72.9	2.2	**76.2**
VGG16	15.36	14.72	70.1	**1.4**	50.3
VGG19	19.52	20.03	69.2	1.6	47.7
Xception	4.55	20.89	70.4	2.3	54.1
ResNet50	3.86	23.61	69.5	2.5	54.1
ResNet101	7.58	42.68	70.1	4.2	48.7
ResNet152	11.29	58.40	71.6	5.6	46.0
DenseNet121	2.83	7.05	71.5	4.4	61.2
DenseNet169	3.36	12.66	70.7	5.7	57.7
DenseNet201	4.29	18.35	70.2	6.5	54.9

*The test accuracies, parameters, and computing operations are expressed in percentages (0–100%), millions of parameters (M), and billions of multiply-accumulates (GMACs), respectively. Training and inference were both performed on a Google Cloud TPU. The EfficientNetB0 network achieved the highest test accuracy, VGG16 the fastest inference time, and MobileNetV2 the best NetScore and least number of parameters and computing operations (bolded)*.

Although the VGG16 and VGG19 networks have the largest number of computations (i.e., ~15.4 and ~19.5 billion multiply-accumulates, respectively), they resulted in the fastest inference times (i.e., on average 1.4 and 1.6 ms per image). In comparison, the DenseNet201 network has 72.1 and 78% fewer operations than VGG16 and VGG19, respectively, but was 364 and 306% slower. These performance trends concur with Ding et al. (2021), who recently showed that (1) the number of computing operations does not explicitly reflect the actual inference speed, and (2) VGG-style architectures can run faster and more efficiently on computing devices compared to more complicated architectures like DenseNets due to their relatively simple designs (i.e., consisting of basic convolutions and ReLU activations). Note that our inference times were calculated on the Cloud TPU using a batch size of 8. The relative inference speeds between the benchmarked CNN architectures (i.e., their ordering from fastest to slowest) may differ across different computing devices given that some platforms are designed to accelerate certain operations better than others (e.g., cloud computing vs. those designed for mobile and embedded systems).

The ResNet152 network achieved one of the highest image classification accuracies on the ExoNet database (71.6% accuracy). However, it received the lowest NetScore (Ω = 46) due to the disproportionally large number of parameters (i.e., containing more parameters than any other benchmarked CNN architecture). Surprisingly, the EfficientNetB0 network did not receive the highest NetScore (Ω = 72.6) despite achieving the highest image classification accuracy on the ExoNet database and the architecture having been designed using a neural architecture search to optimize the classification accuracy and computational complexity. The MobileNetV2 network, which uses depthwise separable convolutions, received the highest NetScore (Ω = 76.2), therein demonstrating the best balance between the image classification accuracy (72.9% accuracy) and the architectural and computational complexities. These results suggests that, of the benchmarked CNN architectures, the MobileNetV2 network might be the most applicable for onboard real-time inference with mobile computing devices, as would be the case for robotic leg prostheses and exoskeletons.

Tables 6–8 show the multiclass confusion matrix for EfficientNetB0, MobileNetV2, and VGG16; the other benchmarked CNN architectures displayed a similar interclass trend. The matrix columns and rows are the predicted and labeled classes, respectively. The diagonal elements are the classification accuracies for each environment class during inference, known as true positives, and the nondiagonal elements are the misclassification percentages; the darker shades represent higher classification accuracies. The networks most accurately predicted the “level-ground transition wall/door” (L-T-W) class with an average accuracy of 84.3 ± 2.1%, followed by the “level-ground steady” (L-S) class with an average accuracy of 77.3 ± 2.1% and the “decline stairs transition level-ground” (D-T-L) class with an average accuracy of 76.6 ± 2.4%. Note that these accuracies, expressed in percentage points, are averages ± one standard deviation across the benchmarked CNN architectures. These results could be attributed to the class imbalances among the training data such that there were significantly more images of L-T-W environments compared to other classes. However, some classes with limited images showed relatively good classification performance. For instance, the “incline stairs transition level-ground” (I-T-L) class includes only 1.2% of the ExoNet database but achieved 71.2% ± 5.1% average classification accuracy. Not surprisingly, the least accurate predictions were for the environment classes that contain “other” features—i.e., the “wall/door transition other” (W-T-O) class with an average accuracy of 38.3 ± 3.5% and the “level-ground transition other” (L-T-O) class with an average accuracy of 46.8 ± 2.4%. These results could be attributed to the greater noise and randomness of the environments and/or objects in the images.

**Table 6:** The multiclass confusion matrix for EfficientNetB0 showing the image classification accuracies (%) during inference on the ExoNet database.

	**D-T-L**	**W-S**	**W-T-O**	**I-S**	**I-T-W**	**I-T-L**	**L-S**	**L-T-D**	**L-T-W**	**L-T-I**	**L-T-O**	**L-T-E**
D-T-L	78.8	0.6	0.3	0.0	0.0	0.0	1.0	4.5	10.6	0.2	2.6	1.4
W-S	0.2	72.1	9.2	0.0	0.3	0.2	0.0	0.3	15.2	0.1	1.9	0.5
W-T-O	0.4	21.9	43.2	0.0	0.4	0.2	0.1	0.1	19.2	0.4	8.8	5.5
I-S	0.0	0.3	0.1	62.1	33.9	1.9	0.0	0.0	0.5	0.6	0.5	0.0
I-T-W	0.0	2.0	0.7	16.8	69.0	2.8	0.0	0.2	1.5	5.8	0.6	0.6
I-T-L	1.0	0.5	0.2	2.5	5.7	77.9	2.2	0.2	6.4	1.5	1.2	0.7
L-S	0.1	0.4	0.0	0.0	0.0	0.1	79.9	0.3	11.9	0.6	6.4	0.2
L-T-D	5.5	0.4	0.6	0.0	0.3	0.1	1.0	53.3	28.3	2.8	4.3	3.6
L-T-W	0.3	1.5	0.4	0.0	0.0	0.1	3.7	0.4	86.5	0.3	4.4	2.3
L-T-I	0.1	1.0	0.2	1.0	3.9	0.4	3.8	0.7	23.3	49.1	12.1	4.4
L-T-O	0.3	0.6	1.1	0.0	0.1	0.1	13.7	0.5	28.8	0.7	47.5	6.6
L-T-E	0.3	0.5	0.3	0.0	0.0	0.1	0.8	0.5	14.0	0.6	10.1	72.7

*The columns and rows are the predicted and labeled classes, respectively*.

**Table 7:** The multiclass confusion matrix for MobileNetV2 showing the image classification accuracies (%) during inference on the ExoNet database.

	**D-T-L**	**W-S**	**W-T-O**	**I-S**	**I-T-W**	**I-T-L**	**L-S**	**L-T-D**	**L-T-W**	**L-T-I**	**L-T-O**	**L-T-E**
D-T-L	78.3	0.6	0.4	0.0	0.1	0.0	0.9	5.0	9.9	0.2	2.9	1.8
W-S	0.2	73.4	10.1	0.0	0.3	0.3	0.1	0.4	13.4	0.2	1.2	0.5
W-T-O	0.6	24.2	41.5	0.0	0.4	0.0	0.0	0.6	18.0	0.6	9.5	4.6
I-S	0.0	0.4	0.1	64.7	28.9	2.6	0.4	0.0	0.9	1.3	0.1	0.6
I-T-W	0.0	2.0	0.9	18.5	66.2	3.3	0.0	0.1	2.7	4.9	0.5	1.0
I-T-L	0.2	0.7	0.0	2.2	7.9	73.6	3.4	0.2	7.9	1.7	1.0	1.3
L-S	0.1	0.3	0.0	0.0	0.0	0.1	79.2	0.1	11.9	0.2	7.7	0.4
L-T-D	5.5	0.7	0.4	0.0	0.0	0.1	1.0	53.8	28.2	1.1	5.5	3.7
L-T-W	0.3	1.9	0.5	0.0	0.0	0.1	3.9	0.4	86.5	0.3	4.1	2.0
L-T-I	0.2	1.2	0.1	0.1	3.9	0.4	4.1	1.0	26.1	48.4	9.5	4.9
L-T-O	0.3	0.4	1.2	0.0	0.0	0.1	13.9	0.6	29.5	0.4	47.9	5.6
L-T-E	0.4	0.5	0.6	0.0	0.1	0.1	0.9	0.3	15.3	0.6	10.7	70.7

*The columns and rows are the predicted and labeled classes, respectively*.

**Table 8:** The multiclass confusion matrix for VGG16 showing the image classification accuracies (%) during inference on the ExoNet database.

	**D-T-L**	**W-S**	**W-T-O**	**I-S**	**I-T-W**	**I-T-L**	**L-S**	**L-T-D**	**L-T-W**	**L-T-I**	**L-T-O**	**L-T-E**
D-T-L	75.5	0.6	0.6	0.0	0.0	0.2	1.1	5.3	9.9	0.0	3.5	3.3
W-S	0.2	66.1	8.0	0.0	0.1	0.2	0.2	0.1	22.3	0.1	1.7	1.0
W-T-O	0.3	23.6	34.2	0.1	0.1	0.2	0.1	0.2	23.5	0.8	10.1	6.7
I-S	0.0	0.4	0.6	59.2	30.1	3.3	1.5	0.0	1.7	0.9	0.5	1.8
I-T-W	0.1	1.8	1.0	15.1	66.5	1.9	0.0	0.1	5.0	5.4	1.4	1.8
I-T-L	0.5	0.0	0.2	4.5	8.1	64.8	3.9	0.0	13.1	1.9	1.5	1.5
L-S	0.1	0.3	0.0	0.0	0.0	0.0	77.0	0.1	14.8	0.3	7.1	0.2
L-T-D	7.3	0.8	0.7	0.0	0.2	0.0	1.0	43.8	33.3	0.6	7.8	4.5
L-T-W	0.3	1.8	0.4	0.0	0.0	0.1	3.8	0.4	86.9	0.2	4.5	1.7
L-T-I	0.7	1.0	0.3	0.7	5.2	0.6	5.6	1.0	29.9	37.2	12.4	5.4
L-T-O	0.3	0.3	0.9	0.1	0.0	0.0	14.7	0.4	35.2	0.5	41.7	5.8
L-T-E	0.6	0.6	0.5	0.0	0.0	0.0	1.0	0.3	19.6	0.3	10.0	67.0

*The columns and rows are the predicted and labeled classes, respectively*.

In summary, our comparative analyses showed that the EfficientNetB0 network achieves the highest image classification accuracy on the ExoNet database during inference; VGG16 achieves the fastest inference time; and MobileNetV2 achieves the best NetScore and has least number of parameters and computing operations. These results can help inform the optimal architecture design or selection depending on the desired performance of an environment classification system for robotic leg prostheses and exoskeletons.

## Discussion

Here we developed an advanced environment classification system for robotic leg prostheses and exoskeletons powered by computer vision and deep learning to predict the oncoming walking environment prior to physical interactions, therein allowing for more accurate and robust automated high-level control decisions. We first reviewed our “ExoNet” database (Laschowski et al., 2020b)—the largest open-source dataset of wearable camera images of walking environments. Unparalleled in both scale and diversity, ExoNet contains ~5.6 million images of indoor and outdoor real-world walking environments, of which ~923,000 images were annotated using a hierarchical labeling architecture. In terms of original contributions, we then trained and tested over a dozen state-of-the-art deep convolutional neural networks on the ExoNet database for image classification and automatic feature engineering. Finally, we quantitatively compared the benchmarked CNN architectures and their environment classification predictions using an operational metric called “NetScore,” which balances the image classification accuracy with the computational and memory storage requirements (i.e., important for onboard real-time inference with mobile computing devices). Overall, this study provides a large-scale benchmark and reference for next-generation environment classification systems for robotic leg prostheses and exoskeletons. Applications could also extend to humanoids, autonomous legged robots, powered wheelchairs, and assistive technologies for persons with visual impairments.

The use of deep convolutional neural networks for this computer vision application was made possible because of the ExoNet database. In addition to being open-source, the large scale and diversity of ExoNet significantly distinguishes itself from all previous research (Table 1). The ExoNet database contains ~923,000 labeled images. In comparison, the previous largest dataset, developed by Varol's research group (Massalin et al., 2018), contained ~402,000 images. Whereas previous datasets included fewer than six environment classes, the most common being level-ground terrain and incline and decline stairs, the ExoNet database uses a novel 12-class hierarchical labeling architecture. These differences can have important practical implications since deep learning requires significant and diverse training data to prevent overfitting and promote generalization (LeCun et al., 2015). Although combining closely related classes in the ExoNet database (e.g., the W-T-O and W-S classes) could improve the classification accuracy, these effects would need to be explored in future research. The quality of our images (1,280 × 720) is also considerably higher than previous datasets (e.g., 224 × 224 and 320 × 240). Lower resolution images have been shown to decrease the classification accuracy of walking environments (Novo-Torres et al., 2019; Da Silva et al., 2020). Although higher resolution images can increase the onboard computational and memory storage requirements, using efficient CNN architectures with fewer computing operations like EfficientNetB0 (Tan and Le, 2019) can allow for processing larger images for relatively similar computational cost. As robotic leg prostheses and exoskeletons begin to transition out of research laboratories and into real-world environments, large-scale and challenging datasets like ExoNet are needed to support the development of next-generation image classification algorithms for environment-adaptive locomotor control systems.

A potential limitation of the ExoNet database is the 2D nature of the environment information. Many researchers have likewise used a wearable RGB camera for passive environment sensing (Krausz and Hargrove, 2015; Diaz et al., 2018; Khademi and Simon, 2019; Laschowski et al., 2019b, 2020b; Novo-Torres et al., 2019; Da Silva et al., 2020; Zhong et al., 2020). Although multiple RGB cameras could be used to capture 3D information (i.e., comparable to how the human visual system uses triangulation for depth perception) (Patla, 1997), each pixel in an RGB image contains only light intensity information. Other researchers have used depth cameras to explicitly capture images containing both light intensity information and distance measurements (Krausz et al., 2015, 2019; Varol and Massalin, 2016; Hu et al., 2018; Massalin et al., 2018; Zhang et al., 2019b,c,d, 2020; Krausz and Hargrove, 2021). These range imaging systems work by emitting infrared light and measuring the light time-of-flight between the camera and oncoming walking environment to calculate distance. Depth sensing can uniquely extract environmental features like step height and width, which can improve the mid-level control of robotic leg prostheses and exoskeletons (e.g., increasing the actuator joint torques to assist with steeper stairs).

Despite these benefits, depth measurement accuracy typically degrades in outdoor lighting conditions (e.g., sunlight) and with increasing distance (Krausz and Hargrove, 2019; Zhang et al., 2019a). Consequently, most environment recognition systems using depth cameras have been tested in controlled indoor environments and/or have had limited capture volumes (i.e., 1–2 m of maximum range imaging) (Krausz et al., 2015, 2019; Varol and Massalin, 2016; Hu et al., 2018; Massalin et al., 2018; Zhang et al., 2020). These systems often require an onboard accelerometer or IMU to transform the 3D environment information from the camera coordinate system into global coordinates (Krausz et al., 2015; Zhang et al., 2019b,c,d, 2020; Krausz and Hargrove, 2021). Furthermore, the application of depth cameras for active environment sensing could require robotic leg prostheses and exoskeletons to have microcontrollers with higher computing power and lower power consumption. In such case, the current embedded systems would need significant modifications to support the onboard real-time processing and classification of depth images, as previously acknowledged by Massalin et al. (2018). These practical limitations motivated our decision to use RGB images for the environment sensing and classification.

Our comparative analyses showed that the EfficientNetB0 network (Tan and Le, 2019) achieves the highest image classification accuracy on the ExoNet database during inference (73.2% accuracy). However, for online environment-adaptive control of robotic leg prostheses and exoskeletons, higher classification accuracies would be desired since even rare misclassifications could cause loss-of-balance and injury. Although we used deep convolutional neural networks that are state-of-the-art in computer vision, the architectures included only feedforward connections, thereby classifying the walking environment frame-by-frame without knowledge of the preceding classification decisions. Moving forward, sequential information over time could be used to improve the image classification accuracy and robustness, especially during steady-state environments. This computer vision technique is analogous to how light-sensitive receptors in the human eye capture dynamic images to control locomotion (i.e., known as optical flow) (Patla, 1997). Sequential data could be classified using majority voting (Wang et al., 2013; Varol and Massalin, 2016; Massalin et al., 2018) or deep learning networks like Transformers (Vaswani et al., 2017) or recurrent neural networks (RNNs) (Zhang et al., 2019b). Majority voting works by storing sequential decisions in a vector and generates a classification prediction based on the majority of stored decisions.

In comparison, recurrent neural networks process sequential data while maintaining an internal hidden state vector that implicitly contains temporal information. Training RNNs can be challenging though, due to exploding and vanishing gradients. Although these networks were designed to learn long-term dependencies, research has shown that they struggle with storing sequential information over long periods (LeCun et al., 2015). To mitigate this issue, RNNs can be supplemented with an explicit memory module like a neural Turning machine or long short-term memory (LSTM) network, therein improving gradient flow. Fu's research group (Zhang et al., 2019b) explored the use of temporal data for environment classification. Sequential decisions from a baseline CNN were fused and classified using a recurrent neural network, LSTM network, majority voting, and a hidden Markov model (HMM). The baseline CNN achieved 92.8% image classification accuracy across five environment classes. Supplementing the baseline network with an RNN, LSTM network, majority voting, and HMM resulted in 96.5, 96.4, 95, and 96.8% classification accuracies, respectively (Zhang et al., 2019b). While sequential data can improve the image classification accuracy of walking environments, these decisions often require longer computation times and thus could impede real-time locomotor control.

For onboard real-time inference, the ideal convolutional neural network would need to achieve high image classification accuracy with minimal parameters, computing operations, and inference time. Accordingly, we quantitatively compared the benchmarked CNN architectures and their environment classification predictions using “NetScore” (Wong, 2018), which balances the image classification accuracy with the computational and memory storage requirements. We showed that MobileNetV2 (Sandler et al., 2018), which uses depthwise separable convolutions, achieves the highest NetScore (Ω = 76.2), therein demonstrating the best balance between the classification accuracy (72.9% accuracy) and the architectural and computational complexities. Researchers previously demonstrated the ability of MobileNetV2 to perform onboard real-time inference on a mobile computing device (i.e., ~75 ms per image on a CPU-powered Google Pixel 1 smartphone) (Sandler et al., 2018). However, our classification system could potentially yield even faster runtimes since (1) the smartphone that we used (i.e., the iPhone XS Max) has an onboard GPU, and (2) we reduced the size of the final densely connected layer of the MobileNetV2 architecture from 1,000 outputs, as originally used for ImageNet, to 12 outputs, for the ExoNet database. Compared to traditional CPUs, GPUs have many more core processors, which permit faster and more efficient CNN computations through parallel computing (LeCun et al., 2015). Moving forward, we recommend using the existing CPU embedded systems in robotic leg prostheses and exoskeletons for locomotion mode recognition based on neuromuscular-mechanical data, which is less computationally expensive, and a supplementary GPU computing device for environment classification; these recommendations concur with those recently proposed by Huang and colleagues (Da Silva et al., 2020).

Finally, future research is needed in multi-sensor data fusion. Since the environmental context does not explicitly represent the user's locomotor intent, data from computer vision should supplement, rather than replace, the automated locomotion mode control decisions based on mechanical, inertial, and/or neuromuscular sensors. Images from our wearable smartphone camera could be fused with its onboard IMU measurements to improve performance. For example, when an exoskeleton or prosthesis user wants to sit down, the acceleration data would indicate stand-to-sit rather than level-ground walking, despite level-ground terrain being accurately detected within the visual field-of-view (e.g., see the bottom right image in Figure 6). Inspired by Zhang et al. (2011), Wang et al. (2013), Diaz et al. (2018), Khademi and Simon (2019), Da Silva et al. (2020), our smartphone IMU measurements could also help minimize the onboard computational and memory storage requirements *via* sampling rate control (i.e., providing an automatic triggering mechanism for the image capture). Whereas faster walking speeds could benefit from higher sampling rates for continuous classification, standing still does not necessarily require environment information and thus the smartphone camera could be powered down or the sampling rate decreased, therein conserving the onboard operating resources. However, relatively few researchers have fused environment data with mechanical and/or inertial measurements for automated locomotion mode recognition (Huang et al., 2011a; Zhang et al., 2011; Du et al., 2012; Wang et al., 2013; Liu et al., 2016; Krausz et al., 2019; Krausz and Hargrove, 2021) and only one study (Zhang et al., 2020) has used such information for online environment-adaptive control of a robotic prosthesis during walking (i.e., stepping over an obstacle). These limitations in systems integration offer exciting challenges and opportunities for future research.

## Data Availability Statement

The original contributions presented in the study are included in the article/supplementary material, further inquiries can be directed to the corresponding author.

## Ethics Statement

Ethical review and approval was not required for the study on human participants in accordance with the local legislation and institutional requirements. The patients/participants provided their written informed consent to participate in this study. Written informed consent was obtained from the individual(s) for the publication of any potentially identifiable images or data included in this article.

## Author Contributions

BL: contributed to the study design, literature review, data collection, image labeling, CNN training and testing, analyses of the results, and manuscript writing. WM: contributed to the study design, code development, CNN training and testing, analyses of the results, and manuscript writing. AW and JM: contributed to the study design, analyses of the results, and manuscript writing. All authors read and approved the final manuscript.

## Funding

This research was funded by BL and WM Ph.D scholarships with the Natural Sciences and Engineering Research Council of Canada (NSERC), BL Waterloo Engineering Excellence Ph.D. fellowship, JM Canada Research Chair in Biomechatronic System Dynamics, and AW Canada Research Chair in Artificial Intelligence and Medical Imaging.

## Conflict of Interest

The authors declare that the research was conducted in the absence of any commercial or financial relationships that could be construed as a potential conflict of interest.

## Publisher's Note

All claims expressed in this article are solely those of the authors and do not necessarily represent those of their affiliated organizations, or those of the publisher, the editors and the reviewers. Any product that may be evaluated in this article, or claim that may be made by its manufacturer, is not guaranteed or endorsed by the publisher.
